# Foliar Herbivory Reduces Rhizosphere Fungal Diversity and Destabilizes the Co-occurrence Network

**DOI:** 10.3389/fmicb.2022.846332

**Published:** 2022-03-08

**Authors:** Yu Shi, Kaoping Zhang, Tiantian Ma, Zhongyue Zhang, Ping Li, Zhenlong Xing, Jianqing Ding

**Affiliations:** ^1^State Key Laboratory of Crop Stress Adaptation and Improvement, School of Life Sciences, Henan University, Kaifeng, China; ^2^Wuhan Botanical Garden, Chinese Academy of Sciences, Wuhan, China

**Keywords:** foliar chewing herbivore, rhizosphere fungal community, *Triadica sebifera*, diversity, assembly processes, stability

## Abstract

Insect herbivores can adversely impact terrestrial plants throughout ontogeny and across various ecosystems. Simultaneously, the effects of foliar herbivory may extend belowground, to the soil microbial community. However, the responses in terms of the diversity, assembly, and stability of rhizosphere fungi to aboveground herbivory remain understudied. Here, using high-throughput sequencing, the effects of foliar insect herbivory on rhizosphere fungal microbes were investigated in a common garden experiment that manipulated herbivory intensity and time from herbivore removal. The number of observed fungal species was reduced by a greater herbivory intensity, with some species evidently sensitive to herbivory intensity and time since herbivore removal. Rhizofungal assembly processes were altered by both herbivory intensity and time since herbivore removal. Further, we found evidence that both factors strongly influenced fungal community stability: a high intensity of herbivory coupled with a shorter time since herbivore removal resulted in low stability. These results suggest that foliar herbivory can adversely alter fungal diversity and stability, which would in turn be harmful for plant health. Fortunately, the effect seems to gradually diminish with time elapsed after herbivore removal. Our findings provide a fresh, in-depth view into the roles of rhizofungi in enhancing the adaption ability of plants under environmental stress.

## Introduction

All plants inevitably face various biotic and abiotic stresses (Bagchi et al., 2014; Kaisermann et al., 2017; Li et al., 2021). As an important biotic stress, insect herbivores may threaten plants in agricultural settings, forests, and other terrestrial ecosystems (Schoonhoven et al., 1998). During herbivore–plant coevolution, plants gained the ability to enhance their interaction with beneficial soil microbes to overcome this problem (Pineda et al., 2010; Tedersoo et al., 2020) by producing various metabolites (Howe and Jander, 2008; McCormick et al., 2012; Mithoefer and Boland, 2012) that can regulate plant–microbe interactions belowground (Biere and Bennett, 2013; Pangesti et al., 2013; Humphrey and Whiteman, 2020). Accordingly, soil microorganisms can be manipulated to help plants counter attacks and fitness impacts from aboveground insect herbivores (Pineda et al., 2017; Jiang et al., 2020). The rhizosphere is a major source of the plant microbiome and is considered as a “second genome” of plants due to its importance for their health (Lu et al., 2018). In particular, fungi are critical participants in soil biochemical cycles and they have been proven crucial for promoting plant growth and health in various environments (Bahram et al., 2013; van der Heijden et al., 2016; Fisher and Garner, 2020; Yuan et al., 2021b). The rhizosphere fungal community may undergo differential responses in the face of aboveground herbivory due to cascading effects spanning from herbivores to plants to the soil (Cai et al., 2016; Ma et al., 2018a). However, how the rhizofungal diversity and community shift under plant stress caused by insect herbivory remains understudied.

Currently, a key unresolved issue in microbial ecology is our understanding of microbial community assembly processes (Zhou et al., 2008; Stegen et al., 2012; Yang, 2021). To the best of our knowledge, microbial assembly can entail the following five processes: dispersal limitation, drift, homogenous selection, heterogeneous selection, and homogenous dispersal; all these scenarios were carefully described by Shi et al. (2019a). These five scenarios have been extensively studied and revealed in natural (Dini-Andreote et al., 2015; Stegen et al., 2015) and agricultural ecosystems (Shi et al., 2018; Dong et al., 2020; Jiao et al., 2020), with literature now available that reports on the response mechanism of assembly processes to long term fermentation (Fan et al., 2017), climate warming (Howe and Jander, 2008), succession of soil (23), and so forth. Yet that body of work has mainly investigated the bacterial community assembly processes in different habitats. For the fungi, there is a paucity of similar research, likely because of their highly divergent phylogenetic tree (Ning et al., 2020). The high sequence variability of the ITS marker gene can lead to more accurate taxonomic identification than is possible with SSU rRNA, but it makes sequence alignment a lot more unreliable (Xue et al., 2019; Ning et al., 2020). Fortunately, an approach named Phylogenetic-Bin-Based Null Model Analysis (iCAMP) provides an opportunity to explore fungal community assembly by using a “ghost” tree based on ITS fungal sequences (Ning et al., 2020), which is more reliable than a fast tree. It was suggested that iCAMP had higher specificity and accuracy than the entire community-based method and provides an effective tool to quantify microbial assembly processes (Ning et al., 2020). Therefore, it is now possible to survey how rhizofungal community assembly processes vary under herbivory stresses using this newly developed iCAMP approach.

Mounting evidence has revealed that species interactions in biological community networks are crucial for their stability (Neutel et al., 2002; Coux et al., 2016). Likewise, the capacity of soil microbial communities to maintaining stability under disturbances largely depends on the complexity of interactions between species within that community (Steele et al., 2011; Schmitt et al., 2012; Wu et al., 2021). Recently, co-occurrence network approaches have been increasingly applied to investigate in detail the association relationships among microbial individuals (Faust et al., 2012; Ma et al., 2018b; Shi et al., 2020a; Yuan et al., 2021a). In addition, network robustness, calculated as the degree of natural connectivity determined by “attacking” the edges and nodes characterizing the network (removing the nodes and edges in the network randomly; Albert et al., 2000; Peng and Wu, 2016) is frequently being used (Fan et al., 2018; Shi et al., 2019b; Wu et al., 2021); higher robustness of networks (greater resistance of natural connectivity under attack) indicates that the community is more stable, while a sharply declining slope indicates lower network stability. It is conceivable that rhizofungal community networks may be sensitive to aboveground insect herbivory, as a form of biotic disturbance; nevertheless, their property changes and stability in response to insect herbivory remain largely unknown.

To investigate how the rhizofungal community, assembly, and stability each responded to insect herbivory, Chinese tallow (*Triadica sebifera* L.) and larvae of *Spodoptera litura* Fabricius (a generalist herbivorous insect that attack Chinese tallow) were selected as a model system (Zhang et al., 2015). Chinese tallow, a perennial tree native to Asia, can produce numerous defensive metabolites, such as tannins and flavonoids, to resist aboveground insect herbivory (Wang et al., 2012; Huang et al., 2014). Recently, studies have reported that root-associated (mycorrhizal) fungi were strongly influenced by the flavonoids in both the roots and root exudates of Chinese tallow (Pei et al., 2020; Tian et al., 2021). These metabolites of *Triadica* plants in roots can be induced by foliar insect herbivory (Xiao et al., 2019). This indicates that plant metabolites may be a bridge between foliar insect herbivores and root-associated microbes. In addition, the self-recovery and self-maintenance of soil microbes after disturbance are important for soil health (Shade et al., 2012; Lehmann et al., 2020). Based on the knowledge above, here, we hypothesized that foliar insect herbivory and their elapse time would affect overall rhizofungal community composition, and we expected that the rhizofungal association network and its stability would be altered.

## Materials and Methods

### Plants and Insects

To investigate the response of the rhizofungal community to foliar herbivory, we used Chinese tallow [*T. sebifera* ‘Small’ (Euphorbiaceae)] and the armyworm *S. litura* (Lepidoptera: Noctuidae; a leaf-eating caterpillar) as our study system. Chinese tallow seeds were collected in November 2018 from a natural population near Wuhan, China (31°33′N, 114°07′E). *Spodoptera litura* larvae were obtained from Henan Jiyuan Baiyun Industry Co., Ltd., and reared on an artificial diet (Tu et al., 2010).

### Experimental Design

*Triadica* seeds were sown in an unheated greenhouse at Henan University, Henan, China (34°49′N, 114°18′E), in April 2019. Four weeks later, similar-sized plants (with four or five leaves on them) were transplanted into individual pots (height: 15 cm, upper diameter: 19 cm, lower diameter: 12 cm). These pots were filled with a commercial soil and topsoil (surface litter was removed and then the topsoil was passed through a 1 cm mesh screen) that came from an abandoned field that had been used to grow tallow trees at Henan University (1:1 v:v). To diminish the space effects, we randomly shifted the position of the plants every day. To protect against natural insect herbivores, we enclosed all the prepared tallow plants within a nylon mesh cage (6 m × 4 m × 2 m, 0.1 mm mesh size). Then, 50-day-old healthy and uniform plants were used for the following herbivory treatments.

To understand the effects of foliar insect herbivory on soil fungi dynamics, we established treatment groups consisting of three different herbivory intensities (no herbivory, NH; low herbivory, LH; and high herbivory, HH) and three different durations of elapsed time since herbivore removal (2 weeks since herbivore removal, 2 W; 4 weeks since herbivore removal, 4 W; and 6 weeks since herbivore removal, 6 W). The herbivory treatments were implemented as follows: LH: One *S. litura* larva was placed on one healthy plant and removed after 7 days of feeding (~10% leaf area consumed); HH: Four *S. litura* larvae were placed on one healthy plant and removed after 7 days of feeding (~60% leaf area consumed). The control group (NH) comprised healthy plants with no herbivores. Each treatment had three replicates. To investigate the effects of time since herbivore removal upon soil fungal communities, we timed our harvests for 2 W, 4 W, and 6 W post-herbivore removal.

### Soil Sampling, DNA Extraction, and Amplicon Sequencing

Holding their shoots, the experimental plants were gently shaken to remove any loosely bound soil, and then the soil that remained tightly bound to the surface of the fine roots was collected by manually brushing the soils and designated the rhizosphere soil (Philippot et al., 2013; Fan et al., 2017). In total, 27 rhizosphere soil samples were collected. All of these samples were stored at −20°C, and their DNA was extracted within 2 weeks.

Using a Power Soil DNA kit (MO BIO, Carlsbad, CA, United States), total DNA from each soil sample was extracted and stored at −40°C. To quantify the DNA concentration, a NanoDrop ND-1000 spectrophotometer (Thermo Scientific, United States) was used; the DNA was diluted to approximately 25 ng/μl using distilled water and stored at −20°C until analysis.

The nuclear ribosomal internal transcribed spacer region (ITS rRNA gene) was amplified by polymerase chain reaction (PCR), using the fungal primer set ITS5-F (5′-GGAAGTAAAAGTCGTAACAAGG-3′) and ITS2-R (5′-GCTGCGTTCTTCATCGATGC-3′; Bokulich and Mills, 2013; Shi et al., 2020b). For the PCR, the samples were initially denatured at 98°C for 1 min, followed by 30 cycles of denaturation at 98°C for 10 s, primer annealing at 50°C for 30 s, and extension at 72°C for 30 s; a final extension step of 5 min at 72°C was added, to ensure complete amplification of the target region. Each sample was amplified in triplicate, and the ensuing PCR products from each sample were pooled and purified. Next, the PCR products from each sample were combined in equimolar ratios in one tube and sequenced using an Illumina NovaSeq 6,000 platform at NovoGene, Beijing, China.^1^

### Rhizosphere Soil Fungal Community Structure Analyses

Using QIIME 2 software (version 2019.7; Bolyen et al., 2019), the raw data sequences were processed and analyzed by following the workflow at https://qiime2.org. Briefly, to obtain the sub-operational taxonomic unit (OTU) table, quality control of the raw sequencing data was performed using the Deblur tool (Amir et al., 2017). To remove low-quality regions from the sequences, each sequence was truncated at 120 bp according to a sequence quality plot. *De novo* chimera filtering by the VSEARCH tool (Rognes et al., 2016) was used to identify and filter out chimeras. Although the high sequence variability of the ITS marker gene can lead to a more accurate taxonomic identification than is possible using SSU rRNA, it also makes sequence alignment a lot more unreliable. To access reliable phylogenetic information from the ITS region, ghost-tree (Fouquier et al., 2016) was used to create a phylogenetic tree. For this, a pre-built version (ghost_tree_90_qiime_ver7_dynamic_10.10.2017) was used and the command “qiime vsearch cluster-features-closed-reference” was applied, to filter the feature table so as to retain those IDs that matched the ghost tree, and nearly 65% of high quality sequences (1,005,160/1,546,429) were retained. The downstream data analysis was based on the retained sequences. Subsequent taxonomy assignment was done using the Sklearn-based taxonomy classifier with the dynamic Unite database from 10 October 2017.^2^ To compare fungal diversity metrics between the six treatments, 7,017 high-quality sequences were randomly selected for each sample.

### Analyzing the Rhizosphere Soil Fungal Assembly Processes

The assembly of rhizosphere soil fungal communities under different foliar herbivory treatments was estimated using iCAMP, which is based on the turnover of each bin across samples (Ning et al., 2020). The five assembly processes—drift (DR), homogenizing dispersal (HD), dispersal limitation (DL), heterogeneous selection (HeS), and homogeneous selection (HoS)—were quantified in this study. Briefly, running the iCAMP procedure entails three major steps: the first is phylogenetic binning (dividing the observed data into different groups, namely “bins”); the second is the bin-based null model analysis to partition deterministic and stochastic contributions to HoS, HeS, HD, DL, and DR; and the third is to robustly integrate the results of different bins to assess the relative importance of each process (Ning et al., 2020). The minimal size of all bins in this study was 12, with 49 fungal bins obtained. A confidence index (percentage of null values less extreme than the observed value, i.e., a non-parametric one-side confidence level) was applied for the null model significance test.

### Analyzing the Rhizosphere Soil Fungal Stability

Following the approach described by Weiss, co-occurrence networks were constructed using the SparCC package (Weiss et al., 2016). To improve the reliability of these networks, the OTU table was first filtered. Here we constructed six networks corresponding to the six treatment groups: NH, LH, HH, 2 W, 4 W, and 6 W. First, any singletons were removed, and then we retained only those OTUs present in more than 1/3 of all samples per treatment. In this way, 228 OTUs in NH, 203 OTUs in LH, 169 OTUs in HH, 187 OTUs in 2 W, 184 OTUs in 4 W, and 188 OTUs in 6 W were ultimately obtained and used to build the networks [following Weiss et al. (2016)].

Robustness testing is a powerful method to measure a network’s stability and has been used to investigate microbial community stability in multiple studies (Fan et al., 2017; Shi et al., 2019a; Wu et al., 2021). To do that in our study, each network natural connectivity was estimated by “attacking” the nodes (May, 1973) or edges (Jordan, 2009) in the SparCC network. In addition, following Banerjee et al. (2018), in our study network hubs, module hubs, and connectors were defined as keystone species (Shi et al., 2020b). To identify these hubs and connectors, the z-score (within-module degree) and c-score (participation coefficient) of each node within each network were calculated according to the method described by Guimera and Amaral (2005). On the basis of threshold values of the within-module degree (z-score) and participation coefficient (c-score) of nodes, those nodes with a z-score > 2.5 and c-score > 0.6 were designated network hubs, while those with a z-score > 2.5 and c-score < 0.6 were defined as module hubs; nodes with a z-score < 2.5 and c-score > 0.6 were deemed connectors; and nodes with a z-score < 2.5 and c-score < 0.6 were denoted as peripherals. The respective roles of these types of node in community networks has been described at length by Shi et al. (Shi et al., 2020b).

## Results

### Rhizosphere Soil Fungal Community Composition

**Figure 1:**
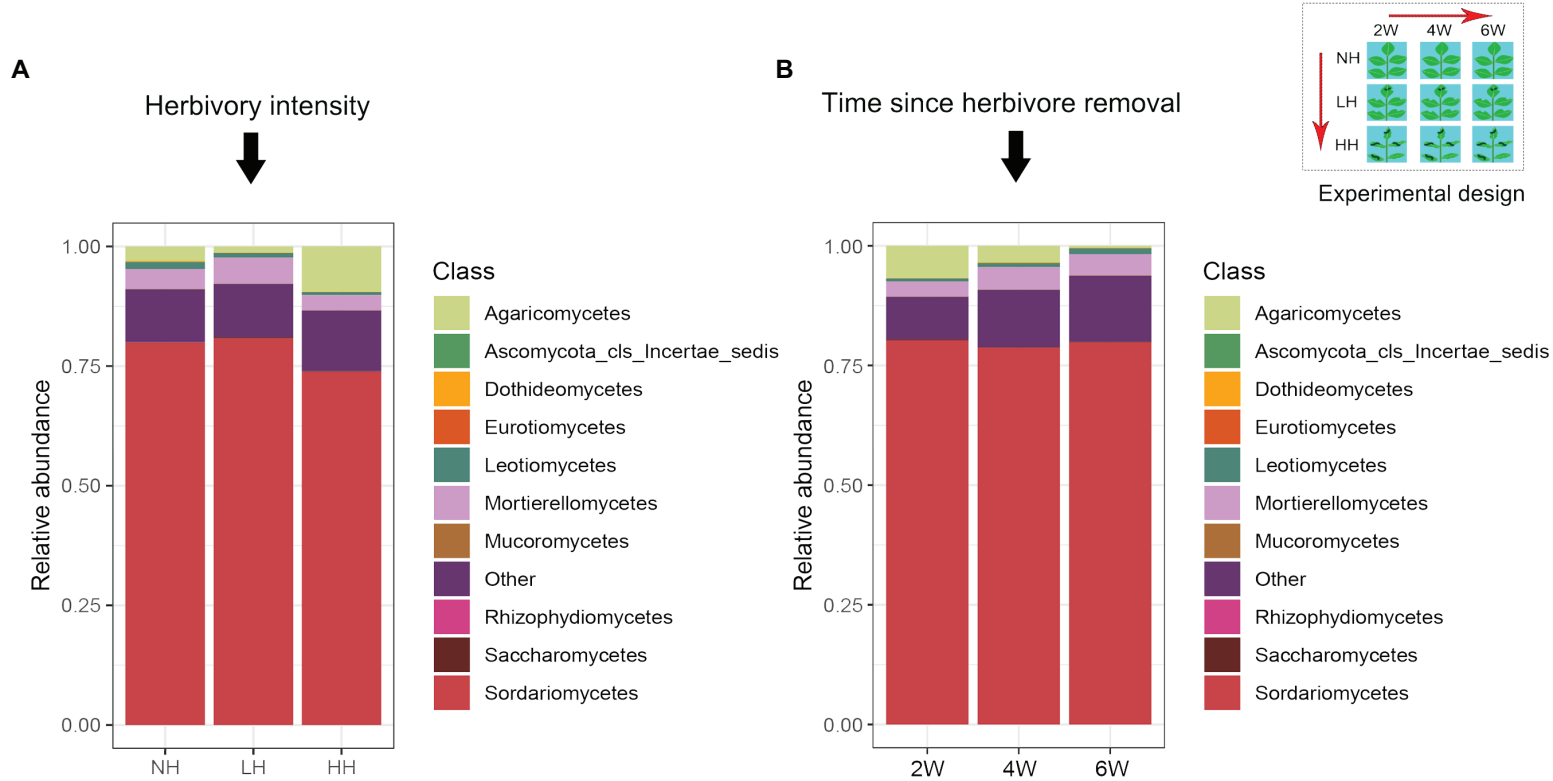
Relative abundance of the dominant fungal classes in rhizosphere soils as affected by different herbivory intensities **(A)** and time since herbivore removal **(B)**.

**Figure 2:**
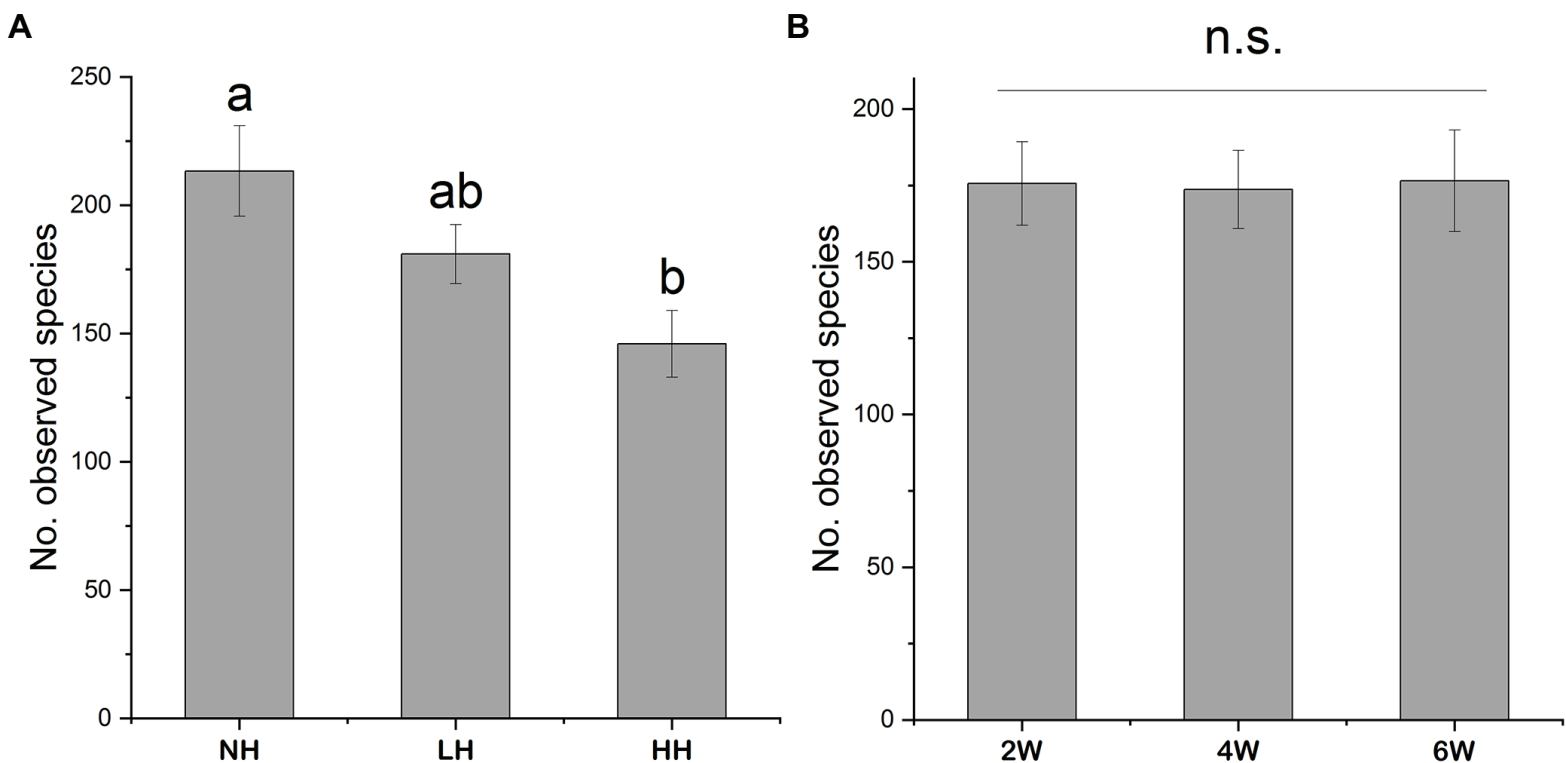
Number of observed rhizosphere fungal operational taxonomic units (OTUs) in rhizosphere soils as affected by different herbivory intensities **(A)** and time since herbivore removal **(B)**. Different letters indicate statistically significant differences between treatments at *p* < 0.05. n.s., non-significant.

Using high-throughput sequencing, we obtained between 7,071 and 44,737 high-quality fungal sequences per sample (mean value: 27,921), of which >99% were classified into 1,569 distinct OTUs. Over 70% of the total OTU sequences belonged to the Acomycotas (Figure 1). The number of observed OTUs was not influenced by the stage of herbivore removal, but it did decrease with increasing herbivory intensity (Figure 2). The soil fungal communities were similar under the differing herbivory intensities and the time since herbivore removal (Table 1). These findings suggested that the rhizosphere soil fungal community was not affected by how long the plants were free of herbivore pressure or the herbivore intensity, but the number of observed species was influenced by herbivory intensity.

**Table 1:** PERMANOVA analysis of the dissimilarity of soil fungal communities as affected by different herbivory intensities and time since herbivore removal.

Treatment	Df	Bray-Curtis		Jaccard	
		Pseudo-F	*p*	Pseudo-F	*p*
Herbivory intensity	2	0.961	0.43	0.968	0.471
Time since herbivore removal	2	1.571	0.114	1.337	0.144
Herbivory intensity × time since herbivore removal	4	1.302	0.191	1.103	0.304

Not only the fungal community as a whole but also the individual species members exhibited variability in sensitivity to time since herbivore removal and herbivore intensity (Table 2). The relative abundance of many species of Agaricomycetes and Sordariomycetes was significantly increased the longer the plants remained free of herbivory. Among the three herbivory intensities, the HH treatment increased the relative abundance of several fungal species and decreased one species in the Agaricales. Furthermore, when compared with LH, the relative abundance of several species in the Ascomycota was decreased by the HH treatment.

**Table 2:** Significantly altered fungal OTUs between treatments, as affected by a different herbivory intensities and time since herbivore removal.

OTU	Up/Down	Taxa
**NH 2W VS NH 4W**		
SH221744.07FU_UDB017890_refs	Down	k__Fungi;p__Basidiomycota;c__Agaricomycetes
SH303020.07FU_UDB017990_refs	Down	k__Fungi;p__Basidiomycota;c__Agaricomycetes
**NH 2W VS NH 6W**		
SH221744.07FU_UDB017890_refs	Down	k__Fungi;p__Basidiomycota;c__Agaricomycetes
SH303020.07FU_UDB017990_refs	Down	k__Fungi;p__Basidiomycota;c__Agaricomycetes
SH220367.07FU_DQ389693_refs	Down	k__Fungi;p__Basidiomycota;c__Agaricomycetes;o__Agaricales;f__Psathyrellaceae;g__Psathyrella
SH183335.07FU_GU212407_reps	Up	k__Fungi;p__Ascomycota;c__Leotiomycetes;o__Helotiales;f__unidentified;g__unidentified;s__unidentified
SH641889.07FU_KU516632_reps	Up	k__Fungi
SH525087.07FU_KP091289_reps	Up	k__Fungi;p__Ascomycota
SH195329.07FU_HM365257_refs	Up	k__Fungi;p__Ascomycota;c__Sordariomycetes;o__Hypocreales;f__Nectriaceae;g__Nectria;s__unidentified
**NH 4W VS NH 6W**		
SH195314.07FU_KP204017_refs	Up	k__Fungi;p__Ascomycota;c__Sordariomycetes;o__Hypocreales;f__Nectriaceae;g__Nectria;s__unidentified
SH183335.07FU_GU212407_reps	Up	k__Fungi;p__Ascomycota;c__Leotiomycetes;o__Helotiales;f__unidentified;g__unidentified;s__unidentified
**LH 2W VS LH 4W**		
SH221744.07FU_UDB017890_refs	Down	k__Fungi;p__Basidiomycota;c__Agaricomycetes
SH303020.07FU_UDB017990_refs	Down	k__Fungi;p__Basidiomycota;c__Agaricomycetes
**LH 2W VS LH 6W**		
SH221744.07FU_UDB017890_refs	Down	k__Fungi;p__Basidiomycota;c__Agaricomycetes
SH303020.07FU_UDB017990_refs	Down	k__Fungi;p__Basidiomycota;c__Agaricomycetes
**LH 4W VS LH 6W**		
None		
**HH 2W VS HH 4W**		
None		
**HH 2W VS HH 6W**		
SH200142.07FU_KU561825_reps	Down	k__Fungi;p__Ascomycota;c__Sordariomycetes;o__Microascales;f__Ceratocystidaceae;g__Ceratocystis
SH179179.07FU_FJ362032_reps	Down	k__Fungi;p__Basidiomycota;c__Agaricomycetes;o__Agaricales
SH346278.07FU_GU237852_refs_singleton	Down	k__Fungi;p__Ascomycota
SH206423.07FU_KU295575_reps	Down	k__Fungi;p__Ascomycota
SH346033.07FU_GU237809_refs	Down	k__Fungi;p__Ascomycota
**HH 4W VS HH 6W**		
SH200142.07FU_KU561825_reps	Down	k__Fungi;p__Ascomycota;c__Sordariomycetes;o__Microascales;f__Ceratocystidaceae;g__Ceratocystis
SH179179.07FU_FJ362032_reps	Down	k__Fungi;p__Basidiomycota;c__Agaricomycetes;o__Agaricales
SH220367.07FU_DQ389693_refs	Down	k__Fungi;p__Basidiomycota;c__Agaricomycetes;o__Agaricales;f__Psathyrellaceae;g__Psathyrella
SH346278.07FU_GU237852_refs_singleton	Down	k__Fungi;p__Ascomycota
SH206423.07FU_KU295575_reps	Down	k__Fungi;p__Ascomycota
SH346033.07FU_GU237809_refs	Down	k__Fungi;p__Ascomycota
**NH 2W VS LH2W**		
NONE		
**NH 2W VS HH2W**		
SH221313.07FU_KT265714_reps	Down	k__Fungi;p__Basidiomycota;c__Agaricomycetes
SH195386.07FU_KP132399_reps	Down	k__Fungi;p__Ascomycota;c__Sordariomycetes;o__Hypocreales;f__Nectriaceae;g__Nectria;s__unidentified
**LH 2W VS HH2W**		
SH179179.07FU_FJ362032_reps	Up	k__Fungi;p__Basidiomycota;c__Agaricomycetes;o__Agaricales
SH346278.07FU_GU237852_refs_singleton	Up	k__Fungi;p__Ascomycota
**NH 4W VS LH4W**		
None		
**NH 4W VS HH4W**		
None		
**LH 4W VS HH4W**		
SH179179.07FU_FJ362032_reps	Up	k__Fungi;p__Basidiomycota;c__Agaricomycetes;o__Agaricales
SH346278.07FU_GU237852_refs_singleton	Up	k__Fungi;p__Ascomycota
SH206423.07FU_KU295575_reps	Up	k__Fungi;p__Ascomycota
SH346033.07FU_GU237809_refs	Up	k__Fungi;p__Ascomycota
SH195774.07FU_AJ244232_refs	Up	k__Fungi;p__Ascomycota;c__Leotiomycetes;o__Helotiales;f__unidentified;g__unidentified;s__unidentified
**NH 6W VS LH6W**		
None		
**NH 6W VS HH6W**		
None		
**LH 6W VS HH6W**		
SH200142.07FU_KU561825_reps	Down	k__Fungi;p__Ascomycota;c__Sordariomycetes;o__Microascales;f__Ceratocystidaceae;g__Ceratocystis		
SH216041.07FU_AF138287_refs	Down	k__Fungi;p__Ascomycota

### Rhizosphere Soil Fungal Assembly Processes

**Figure 3:**
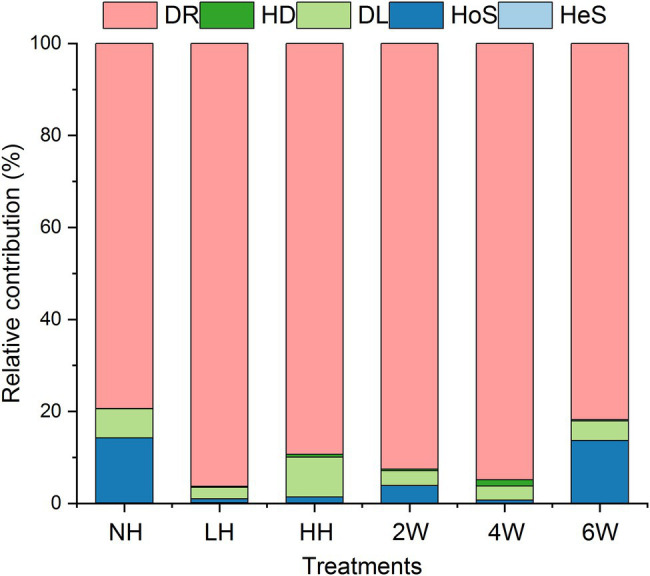
Relative importance of different ecological processes in response to foliar herbivory.

The iCAMP analysis revealed that drift was the most important process among the five responsible for rhizosphere fungal assembly, with an average relative importance of 79.4%–96.3% (Figure 3; Supplementary Table S1). However, foliar herbivory evidently altered the relative importance of the five assembly processes. The LH and HH treatments both increased the relative importance of drift and decreased the ratio of homogeneous selection, and HH also increased the ratio of dispersal limitation. Interestingly, the ratio of homogeneous selection was enhanced under the 6 W treatment; this suggested that how processes contribute to fungal assembly depends on the time elapsed since herbivore removal. In short, both insect herbivory and times since herbivore removal could alter the relevance of the five processes for rhizosphere soil fungal assembly.

**Figure 4:**
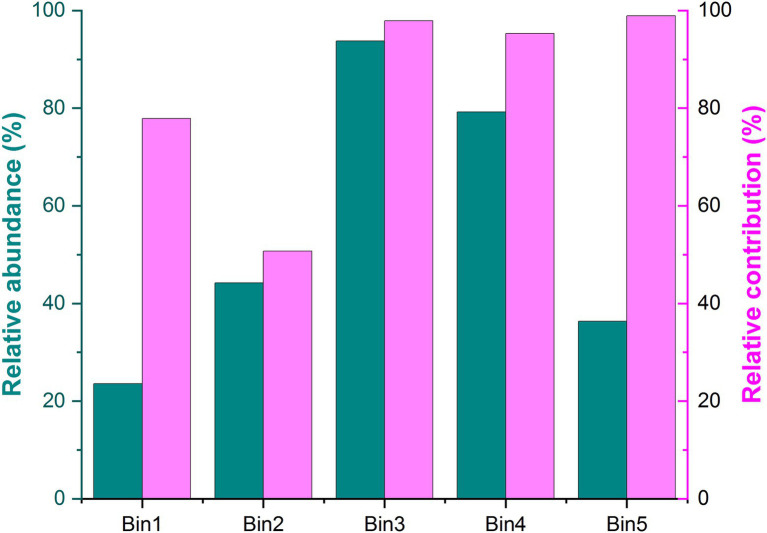
Relative abundance and relative contribution of each bin. Only the five most abundant bins are shown in this figure. Source data can be found in Supplementary Table S3.

**Figure 5:**
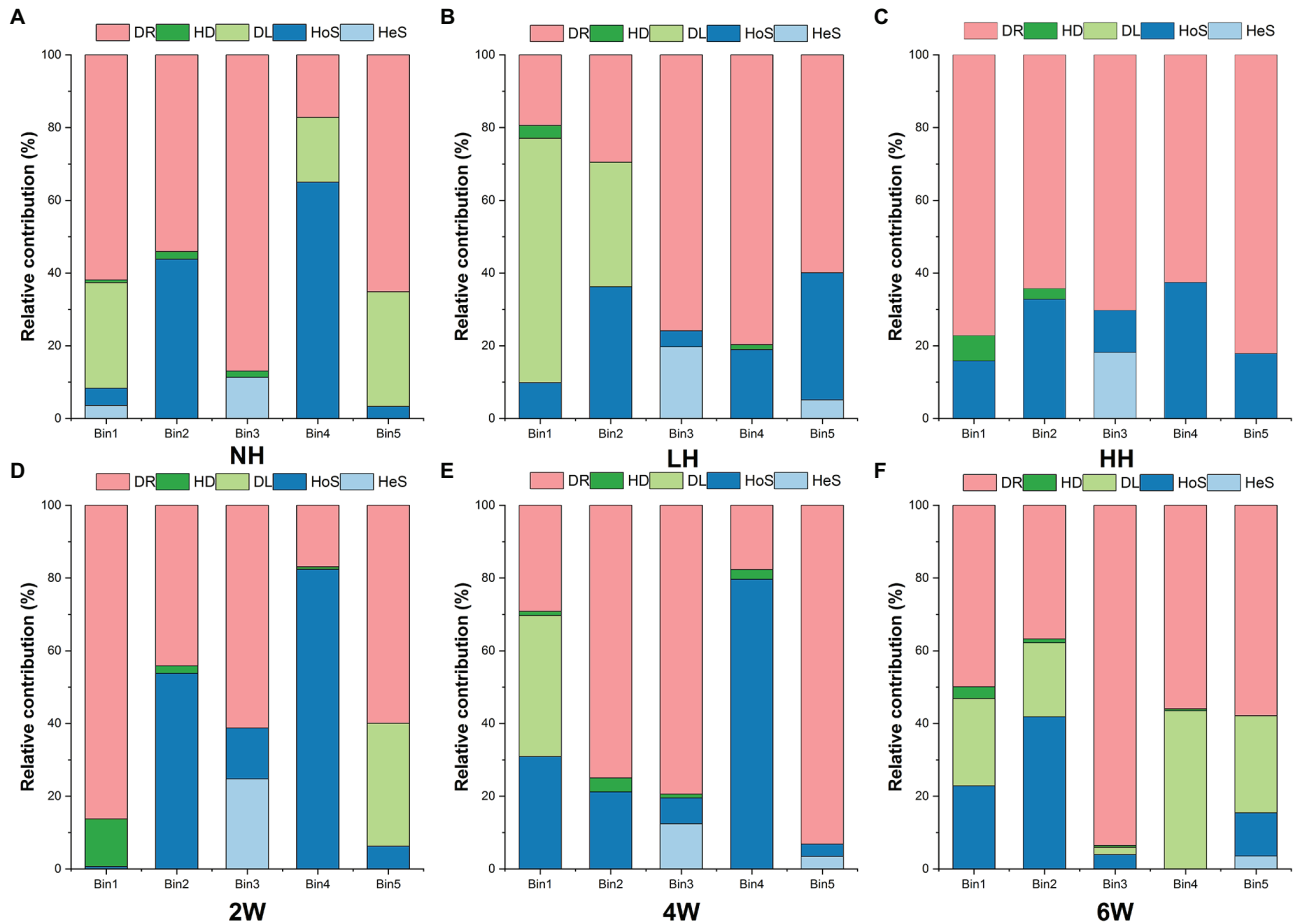
Relative abundance of each bin across the different treatment groups **(A**-**C**: different herbivory intensities; **D**-**F**: different time since herbivore removal). Only the five most abundant bins are shown in this figure. Source data can be found in Supplementary Table S4.

Using iCAMP enabled us to better detect and infer the assembly mechanisms, by providing information on the relative importance of different ecological processes in individual lineages (i.e., bins). Here, the observed 1,569 OTUs were divisible into 49 phylogenetic bins (Supplementary Table S2), yet we found that the relative importance of a given assembly process was independent of the relative abundance in bins (Figure 4). Our results also revealed that the relative importance of each bin not only differed within the same treatment but also among the treatments (Figure 5; Supplementary Table S3).

### Rhizosphere Soil Fungal Stability

**Figure 6:**
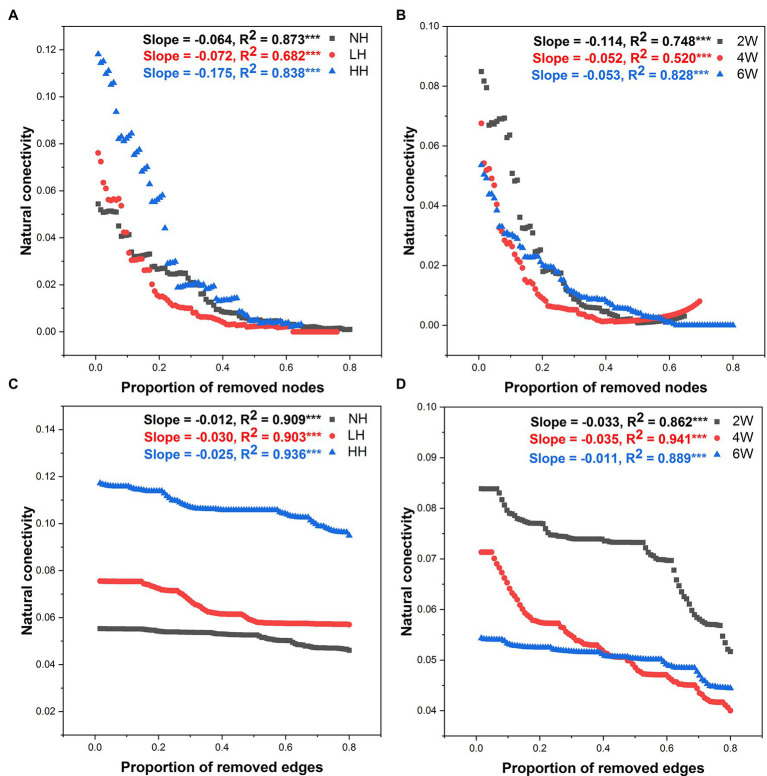
Robustness analysis for the rhizosphere soil fungal community as affected by different lengths of elapsed time since herbivore removal **(A,C)** and differing intensities of herbivory **(B,D)**. Robustness analysis is depicted as the relationships between microbial natural connectivity and the proportion of excluded nodes and edges, such that larger shifts for the same proportion indicate less robustness or stability within the fungal networks. *** *p* < 0.001.

Robustness analysis is a powerful way to test a network’s stability. Accordingly, robustness of fungal networks to the treatments was also tested here, by altering the amplitude of natural connectivity *via* the deletion of nodes and edges (Figure 6). Nodes and edges were discarded in declining order of nodes’ betweenness. Doing this, we found that the natural connectivity of fungal networks presented sharp slopes in HH and 2 W treatment samples, indicating they had weak stability (Figure 6).

To identify the keystone species, z-scores and c-scores were calculated for the nodes in the network for each treatment. There were 4, 2, and 1 keystone species in the NH, LH, and HH treatment groups, respectively, and 7, 0, and 2 keystone species in the 2 W, 4 W, and 6 W treatment groups, respectively (Supplementary Table S4). This pattern suggested foliar insect herbivory as well as time since herbivore removal could slightly reduce the number of keystone species present in the soil fungal community of tallow plants.

## Discussion

### Foliar Herbivory Impact on Rhizofungal Community Composition

In contrast to our hypothesis, herbivory intensity and elapsed time since herbivore removal did not significantly influence the fungal community dissimilarity. This suggests the rhizosphere fungal community may not sensitive to herbivory from leaf-eating insects. However, the fungi are sensitive to partial defoliation is evident by the shift upwards or downwards seen in certain fungal species under the experimentally imposed herbivory conditions. In particular, the number of observed rhizofungal species was found to be significantly lower in the HH than the NH and LH treatments. A previous study showed that aboveground herbivory could affect soil pH (Custer et al., 2020). In addition, myriad studies have reported that the microbial community is strongly related to soil pH (Chu et al., 2010; Fierer, 2017; Xue et al., 2018; Gong et al., 2019). Therefore, the lower number of observed species in HH treatment may be related to altered soil pH, indirectly caused by foliar insect herbivory. We thus suspect that insect herbivory aboveground would enhance root exudation levels, and this could lead to a changed rhizosphere soil pH that influences fungal diversity (Rudrappa et al., 2008; Hoysted et al., 2018).

### Foliar Herbivory Impact on Rhizofungal Assembly Process

Although the drift process drove most of the variation in fungal community composition across the samples from all treatments, slight changes in homogenous selection were discernible in some samples. For example, LH and HH treatments both reduced the relative contribution of the HoS process, while the DL process increased under HH. HoS refers to selection occurring under homogeneous abiotic and biotic conditions in both space and time (Dini-Andreote et al., 2015; Ning et al., 2020). So far as we know, deterministic processes (including HoS) which are mainly driven by biotic and abiotic factors are crucial for microbial community structure, therefore, the disturbed HoS process probably result from altered rhizosphere environments by foliar herbivory (Fan et al., 2017; Zhalnina et al., 2018; Shi et al., 2019b). This suggests herbivory has the potential for reducing the rhizosphere selection ability and limiting the community-level dispersal. This might be harmful for plant health, because the growth of plants is strongly related to their recruited microbes, and diminishing their dispersal ability would likely inhibit their recruitment ability (Bakker et al., 2018). Moreover, we found that the relative contribution of HoS increased at 6 W after herbivore removal. Probably, rhizosphere fungi possess self-regulatory ability after herbivory, which might be helpful for plant growth.

The iCAMP approach let us gain insight into the contribution of each fungal group by bin. Our results suggested that the contribution of each phylum or class in each bin was independent of their relative abundance (Figure 4), and the bin groupings contributed differentially to the five assembly scenarios in each treatment. Although other research has focused on the relative contribution of these processes to variation in soil microbial communities, they often neglect the critical role of each phylum or class. But, the role of these species is very important, because it is beneficial to supplement our knowledge of how to manage key microbial assembly processes by mediating the activity of the highly-contributing microbes (Ning et al., 2020).

### Foliar Herbivory Impact on Rhizofungal Network Stability

Microbial community stability is crucial to ensure ecosystem functioning (Wu et al., 2021). Our study found that aboveground herbivores were quite capable of disturbing rhizofungal community stability. In particular, a high herbivory level significantly reduced the network stability, and this may portend a certain loss of rhizosphere ecological functioning. Further, the stability of the rhizofungal community was lowest at 2 W post-herbivory, and it tended to be stable at 6 W after herbivore removal. This result implied that the plant rhizosphere might exercise a self-recovery function. Numerous studies indicate microbial community stability can be affected by one or more local environmental stresses. For instance, Wu et al. (2021) found that enhanced warming could significantly reduce the microbial stability in the active layer, and Fan et al. found lower network stability in the microbial network of the rhizosphere than in bulk soil (Fan et al., 2017); however, few studies have considered the possibility of temporal or lag effects of biotic or abiotic disturbances to plants. This is the first study we know of that tested rhizofungal community stability under different herbivory intensities and at differing times after herbivore removal from the host plants. Given that plant roots have ability in filtering rhizosphere microbial communities and root exudation can mediate plant-microbe interactions (Fan et al., 2017; Zhalnina et al., 2018), while the root traits can be altered by aboveground herbivory (Karssemeijer et al., 2020), we suspect that foliar herbivory impacts on rhizofungal community network may mediated by the cascading effects on plants (Friman et al., 2021).

### Concluding Remarks

Collectively, our findings reveal that leaf herbivory from an aboveground insect herbivore can affect rhizofungal diversity, with potential weakening of fungal community stability under high herbivory intensity and soon after herbivore removal. The reduced fungal diversity and stability and disrupted assembly processes due to foliar herbivory may impose detrimental effects on plant health in various systems. However, these detrimental effects could be diminished with the extension of herbivore removal, it means the rhizofungal community may possess a self-recovery ability. Our results indicate that aboveground insect herbivores not only influence the rhizosphere microbial community diversity and stability, but might also trigger cascading effects on rhizosphere ecosystem function and cause diminished vitality in this active region belowground. Foliar herbivory should therefore be taken into consideration in future field and greenhouse studies that manipulate soil microbes to improve plant health. Terrestrial ecosystem includes a vast variety of herbivore guilds, plant species and soil types, whether foliar herbivores trigger similar fungi responses in other systems remain unclear, and needs to be further investigated in future studies.

## Data Availability Statement

The datasets presented in this study can be found in online repositories. The names of the repository/repositories and accession number(s) can be found at: https://www.ncbi.nlm.nih.gov/, PRJNA753601.

## Author Contributions

ZX designed the study. YS, ZX, KZ, and TM performed research and analyzed data. YS and ZX wrote the manuscript. ZZ and PL edited the manuscript. JD, YS, and ZX finalized the manuscript. All authors contributed to the article and approved the submitted version.

## Funding

This work was supported by the National Key Research and Development Program of China (2021YFD1900100), the National Natural Science Foundation of China (31770414), and the Natural Science Foundation of Henan (212300410114 and 222300420035).

## Conflict of Interest

The authors declare that the research was conducted in the absence of any commercial or financial relationships that could be construed as a potential conflict of interest.

## Publisher’s Note

All claims expressed in this article are solely those of the authors and do not necessarily represent those of their affiliated organizations, or those of the publisher, the editors and the reviewers. Any product that may be evaluated in this article, or claim that may be made by its manufacturer, is not guaranteed or endorsed by the publisher.

## Footnotes

^1^ www.novogene.com

^2^ https://unite.ut.ee/

## Abbreviations

NH: No herbivory
LH: Low herbivory
HH: High herbivory
2 W: 2 Weeks after herbivore removal
4 W: 4 Weeks after herbivore removal
6 W: 6 Weeks after herbivore removal
DR: Drift
HD: Homogenizing dispersal
DL: Dispersal limitation
HeS: Heterogeneous selection
HoS: Homogeneous selection
