# Supplementary material for: Foliar Herbivory Reduces Rhizosphere Fungal Diversity and Destabilizes the Co-occurrence Network
Source: Front Microbiol. 2022 Mar 8;13:846332. doi: 10.3389/fmicb.2022.846332 (PMC8957981; doi:10.3389/fmicb.2022.846332)
Supplement: Supplementary file 1 [file Table_1.DOCX]

Supplementary Material

for

**Foliar herbivory reduces rhizosphere fungal diversity and destabilizes the co-occurrence network**

Yu Shi^1^, Kaoping Zhang^2^, Tiantian Ma^1^, Zhongyue Zhang^1^, Ping Li^1^, Zhenlong Xing^1*^, Jianqing Ding^1^

^1^State Key Laboratory of Crop Stress Adaptation and Improvement, School of Life Sciences, Henan University, Kaifeng 475004, Henan, China

^2^Wuhan Botanical Garden, Chinese Academy of Sciences, Wuhan 430074, Hubei, China.

*Author for correspondence:

Zhenlong Xing, School of Life Sciences, Henan University, Kaifeng 475004, Henan, China. Email: [zxing@vip.henu.edu.cn](mailto:zxing@vip.henu.edu.cn)

**The following Supporting Information is included for this article:**

**Supplement for table Legends**

Table S1 Relative importance of different ecological processes in response to foliar herbivory.

Table S2 Relative abundance and relative contribution of each bin.

Table S3 Relative abundance of each bin across the different treatment groups of both factors.

Table S4 Keystone species in the different treatments.

Table S1 Relative importance of different ecological processes in response to foliar herbivory.

| **Method** | **GroupBasedOn** | **Group** | **HeS** | **HoS** | **DL** | **HD** | **DR** |
| --- | --- | --- | --- | --- | --- | --- | --- |
| CbMPDiCbraya | herbivory | HH | 0.00081 | 0.013088481 | 0.086868747 | 0.005792635 | 0.893440278 |
| CbMPDiCbraya | herbivory | LH | 0.001353 | 0.008817206 | 0.024895081 | 0.002010635 | 0.96292446 |
| CbMPDiCbraya | herbivory | NH | 0.000717 | 0.141516376 | 0.063421556 | 0.000679381 | 0.793665589 |
| CbMPDiCbraya | duration | 2W | 0.000679 | 0.038486779 | 0.032080866 | 0.003058192 | 0.925694891 |
| CbMPDiCbraya | duration | 4W | 0.000421 | 0.00692975 | 0.030417849 | 0.013536116 | 0.948695371 |
| CbMPDiCbraya | duration | 6W | 0.000583 | 0.135974955 | 0.042372841 | 0.002938943 | 0.818130332 |

Table S2 Relative abundance and relative contribution of each bin.

| Bin | BinRA | TopTaxonID | TopTaxonRAinBin | TopTaxon.Phylum | TopTaxon.Class | Phylum.maxNamed | Phylum.maxNamed.Percent | Class.maxNamed | Class.maxNamed.Percent |
| --- | --- | --- | --- | --- | --- | --- | --- | --- | --- |
| Bin1 | 0.000276157 | SH175573.07FU_JN194729_reps | 0.235887473 | Others | Others | Others | 0.719050169 | Others | 0.778832378 |
| Bin2 | 0.001462217 | SH176586.07FU_JN205828_refs | 0.442096578 | Others | Others | Others | 0.501129352 | Others | 0.50711695 |
| Bin3 | 0.000953804 | SH201993.07FU_EF568042_refs | 0.937848743 | Ascomycota | Others | Ascomycota | 0.980853517 | Others | 0.978861339 |
| Bin4 | 0.000948788 | SH217942.07FU_HM148081_refs | 0.7924098 | Others | Others | Others | 0.899288581 | Others | 0.952986296 |
| Bin5 | 9.56E-05 | SH431739.07FU_AY129287_refs | 0.363763461 | Ascomycota | Leotiomycetes | Ascomycota | 0.98933676 | Leotiomycetes | 0.98933676 |
| Bin6 | 0.000425795 | SH413566.07FU_KF741995_refs | 0.215387711 | Ascomycota | Others | Ascomycota | 1 | Others | 0.947543577 |
| Bin7 | 0.002280705 | SH195604.07FU_GU566286_reps | 0.92084759 | Ascomycota | Others | Ascomycota | 0.999455398 | Others | 0.994894933 |
| Bin8 | 0.000402244 | SH213525.07FU_KP858999_reps | 0.217901904 | Ascomycota | Sordariomycetes | Ascomycota | 1 | Sordariomycetes | 0.647888959 |
| Bin9 | 0.03246242 | SH209070.07FU_EF568081_refs | 0.804360964 | Ascomycota | Sordariomycetes | Ascomycota | 1 | Sordariomycetes | 1 |
| Bin10 | 0.004350269 | SH207979.07FU_AB540575_refs | 0.858611559 | Ascomycota | Sordariomycetes | Ascomycota | 1 | Sordariomycetes | 0.993872373 |
| Bin11 | 0.002323121 | SH175276.07FU_KF494828_reps | 0.574154458 | Ascomycota | Sordariomycetes | Ascomycota | 1 | Sordariomycetes | 0.996445795 |
| Bin12 | 0.013464014 | SH443358.07FU_AY213653_refs | 0.64098659 | Ascomycota | Sordariomycetes | Ascomycota | 1 | Sordariomycetes | 0.99956801 |
| Bin13 | 0.00058407 | SH185946.07FU_JF432973_reps | 0.45379068 | Ascomycota | Sordariomycetes | Ascomycota | 1 | Sordariomycetes | 0.972120912 |
| Bin14 | 0.001047355 | SH175278.07FU_KM231823_refs | 0.420020025 | Ascomycota | Sordariomycetes | Ascomycota | 1 | Sordariomycetes | 1 |
| Bin15 | 0.002362562 | SH554783.07FU_KR909405_refs | 0.544450755 | Ascomycota | Sordariomycetes | Ascomycota | 1 | Sordariomycetes | 0.997813359 |
| Bin16 | 0.002796421 | SH216041.07FU_AF138287_refs | 0.553630532 | Ascomycota | Others | Ascomycota | 1 | Others | 0.784631349 |
| Bin17 | 0.000526835 | SH331396.07FU_KC411761_refs | 0.238838177 | Ascomycota | Others | Ascomycota | 1 | Others | 1 |
| Bin18 | 0.000529522 | SH407683.07FU_AY373907_refs | 0.325036914 | Ascomycota | Others | Ascomycota | 1 | Others | 1 |
| Bin19 | 0.00022412 | SH191316.07FU_AF388928_refs | 0.299952413 | Ascomycota | Others | Ascomycota | 1 | Others | 0.712469049 |
| Bin20 | 0.000261881 | SH215543.07FU_FN397288_reps | 0.17504837 | Ascomycota | Dothideomycetes | Ascomycota | 1 | Dothideomycetes | 0.749545545 |
| Bin21 | 0.007532024 | SH217806.07FU_JX489812_reps | 0.888318618 | Ascomycota | Others | Ascomycota | 0.998448579 | Others | 0.986523664 |
| Bin22 | 0.001383895 | SH247414.07FU_AF328552_refs | 0.522032785 | Ascomycota | Sordariomycetes | Ascomycota | 1 | Sordariomycetes | 0.999333638 |
| Bin23 | 0.720338291 | SH195293.07FU_HM365253_refs | 0.738336335 | Ascomycota | Sordariomycetes | Ascomycota | 0.999969917 | Sordariomycetes | 0.999290321 |
| Bin24 | 0.006440651 | SH202944.07FU_HG328072_reps | 0.903312582 | Ascomycota | Sordariomycetes | Ascomycota | 0.998874457 | Sordariomycetes | 0.940091647 |
| Bin25 | 0.001364246 | SH389464.07FU_HM055443_refs | 0.506010583 | Ascomycota | Sordariomycetes | Ascomycota | 1 | Sordariomycetes | 0.52784485 |
| Bin26 | 0.000550194 | SH212427.07FU_AJ292432_refs | 0.536830313 | Ascomycota | Sordariomycetes | Ascomycota | 1 | Sordariomycetes | 0.637724141 |
| Bin27 | 0.005865533 | SH220565.07FU_GU973628_reps | 0.864495359 | Ascomycota | Sordariomycetes | Ascomycota | 0.955284433 | Sordariomycetes | 0.954810904 |
| Bin28 | 0.000717992 | SH213543.07FU_AB540567_refs | 0.317147443 | Ascomycota | Sordariomycetes | Ascomycota | 0.932714597 | Sordariomycetes | 0.59342423 |
| Bin29 | 0.009376734 | SH527845.07FU_KT596812_reps | 0.364799897 | Ascomycota | Sordariomycetes | Ascomycota | 0.993706065 | Others | 0.575525954 |
| Bin30 | 0.00043575 | SH127902.07FU_DQ780364_refs | 0.598480608 | Ascomycota | Others | Ascomycota | 1 | Others | 0.996258977 |
| Bin31 | 0.000233795 | SH207896.07FU_HQ667522_refs | 0.191669584 | Ascomycota | Others | Ascomycota | 0.991696482 | Leotiomycetes | 0.609830993 |
| Bin32 | 0.081927232 | SH213730.07FU_KU702674_reps | 0.869603312 | Ascomycota | Others | Ascomycota | 0.999899847 | Others | 0.943845823 |
| Bin33 | 0.00117781 | SH174245.07FU_GU721359_refs | 0.714521847 | Ascomycota | Others | Ascomycota | 0.914861136 | Others | 0.979198206 |
| Bin34 | 0.001745349 | SH346278.07FU_GU237852_refs_singleton | 0.514629089 | Ascomycota | Others | Ascomycota | 0.999066002 | Others | 1 |
| Bin35 | 0.000353911 | SH187565.07FU_HE792910_refs | 0.357880512 | Others | Others | Others | 0.583135109 | Others | 1 |
| Bin36 | 0.005928295 | SH198997.07FU_KR232527_reps | 0.975934955 | Ascomycota | Others | Ascomycota | 0.995630879 | Others | 0.995008579 |
| Bin37 | 0.004927212 | SH183333.07FU_JX270594_reps | 0.471159093 | Ascomycota | Leotiomycetes | Ascomycota | 1 | Leotiomycetes | 0.999664002 |
| Bin38 | 0.000556182 | SH212690.07FU_FN397382_reps | 0.297320162 | Ascomycota | Leotiomycetes | Ascomycota | 1 | Leotiomycetes | 0.569250802 |
| Bin39 | 0.00039981 | SH206199.07FU_GU723413_reps | 0.379867135 | Others | Others | Ascomycota | 0.620132865 | Others | 0.964384897 |
| Bin40 | 0.007562871 | SH180118.07FU_HG935614_reps | 0.656814762 | Mortierellomycota | Mortierellomycetes | Mortierellomycota | 0.940517675 | Mortierellomycetes | 0.940517675 |
| Bin41 | 0.036512923 | SH103825.07FU_HQ630301_refs | 0.559149125 | Mortierellomycota | Mortierellomycetes | Mortierellomycota | 0.993079102 | Mortierellomycetes | 0.993079102 |
| Bin42 | 0.000173324 | SH521670.07FU_KU232314_reps | 0.493268593 | Ascomycota | Others | Ascomycota | 0.927576857 | Others | 0.853649055 |
| Bin43 | 0.000226714 | SH640048.07FU_LC096906_reps | 0.319550919 | Basidiomycota | Agaricomycetes | Basidiomycota | 0.946452068 | Agaricomycetes | 0.946452068 |
| Bin44 | 0.000117106 | SH433653.07FU_AF444542_refs | 0.249523311 | Others | Others | Basidiomycota | 0.526027686 | Others | 0.705013133 |
| Bin45 | 5.71E-05 | SH214818.07FU_AB506948_reps | 0.184828081 | Basidiomycota | Others | Basidiomycota | 0.901741466 | Agaricomycetes | 0.716913385 |
| Bin46 | 0.000241295 | SH236790.07FU_AF444438_refs | 0.171773099 | Others | Others | Others | 0.794978184 | Others | 0.880411463 |
| Bin47 | 4.11E-05 | SH197301.07FU_HM008934_refs | 0.307795183 | Basidiomycota | Agaricomycetes | Basidiomycota | 0.722922669 | Agaricomycetes | 0.722922669 |
| Bin48 | 0.035466773 | SH179179.07FU_FJ362032_reps | 0.38567341 | Basidiomycota | Agaricomycetes | Basidiomycota | 0.999687425 | Agaricomycetes | 0.99907958 |
| Bin49 | 5.68E-04 | SH201127.07FU_HG936618_reps | 0.331817183 | Others | Others | Others | 0.965676782 | Others | 0.992850571 |

Table S3 Relative abundance of each bin across the different treatment groups of both factors.

| Method | GroupBasedOn | Group | Index | bin1 | bin2 | bin3 | bin4 | bin5 | bin6 |
| --- | --- | --- | --- | --- | --- | --- | --- | --- | --- |
| CbMPDiCbraya | herbivory | HH | HeS | 0 | 0 | 0.181417381 | 0 | 0 | 0 |
| CbMPDiCbraya | herbivory | HH | HoS | 0.158416038 | 0.327872422 | 0.116006007 | 0.374335046 | 0.17852076 | 0.530056668 |
| CbMPDiCbraya | herbivory | HH | DL | 0 | 0 | 0 | 0 | 0 | 0 |
| CbMPDiCbraya | herbivory | HH | HD | 0.069593594 | 0.029367541 | 0 | 0 | 0 | 0.065132859 |
| CbMPDiCbraya | herbivory | HH | DR | 0.771990369 | 0.642760037 | 0.702576612 | 0.625664954 | 0.82147924 | 0.404810473 |
| CbMPDiCbraya | herbivory | HH | DominantProcess | DR | DR | DR | DR | DR | HoS |
| CbMPDiCbraya | herbivory | HH | DominantProcessImportance | 0.771990369 | 0.642760037 | 0.702576612 | 0.625664954 | 0.82147924 | 0.530056668 |
| CbMPDiCbraya | herbivory | HH | DominantProcessPvalue | 0.134 | 0.328 | 0.111 | 0.281 | 0.127 | 0.378 |
| CbMPDiCbraya | herbivory | LH | HeS | 0 | 0 | 0.197175466 | 0 | 0.050828792 | 0 |
| CbMPDiCbraya | herbivory | LH | HoS | 0.098604152 | 0.362260282 | 0.044018237 | 0.189054002 | 0.349956867 | 0.691748747 |
| CbMPDiCbraya | herbivory | LH | DL | 0.671959896 | 0.342643915 | 0 | 0 | 0 | 0.104150292 |
| CbMPDiCbraya | herbivory | LH | HD | 0.035648593 | 0 | 0 | 0.014260521 | 0 | 0.00851765 |
| CbMPDiCbraya | herbivory | LH | DR | 0.193787359 | 0.295095803 | 0.758806298 | 0.796685477 | 0.599214341 | 0.195583311 |
| CbMPDiCbraya | herbivory | LH | DominantProcess | DL | HoS | DR | DR | DR | HoS |
| CbMPDiCbraya | herbivory | LH | DominantProcessImportance | 0.671959896 | 0.362260282 | 0.758806298 | 0.796685477 | 0.599214341 | 0.691748747 |
| CbMPDiCbraya | herbivory | LH | DominantProcessPvalue | 0.345 | 0.635 | 0.05 | 0.063 | 0.222 | 0.116 |
| CbMPDiCbraya | herbivory | NH | HeS | 0.03584084 | 0 | 0.113271236 | 0 | 0 | 0 |
| CbMPDiCbraya | herbivory | NH | HoS | 0.047464678 | 0.437767322 | 0 | 0.649830184 | 0.033146845 | 0.474058627 |
| CbMPDiCbraya | herbivory | NH | DL | 0.289815558 | 0 | 0 | 0.177865599 | 0.315268826 | 0.045372481 |
| CbMPDiCbraya | herbivory | NH | HD | 0.007980332 | 0.021346299 | 0.016919086 | 0 | 0 | 0.018924187 |
| CbMPDiCbraya | herbivory | NH | DR | 0.618898592 | 0.54088638 | 0.869809678 | 0.172304217 | 0.651584329 | 0.461644704 |
| CbMPDiCbraya | herbivory | NH | DominantProcess | DR | DR | DR | HoS | DR | HoS |
| CbMPDiCbraya | herbivory | NH | DominantProcessImportance | 0.618898592 | 0.54088638 | 0.869809678 | 0.649830184 | 0.651584329 | 0.474058627 |
| CbMPDiCbraya | herbivory | NH | DominantProcessPvalue | 0.212 | 0.412 | 0.007 | 0.156 | 0.132 | 0.533 |
| CbMPDiCbraya | duration | 2W | HeS | 0 | 0 | 0.247355935 | 0 | 0 | 0 |
| CbMPDiCbraya | duration | 2W | HoS | 0.006341984 | 0.537931234 | 0.140445459 | 0.82385789 | 0.06230359 | 0.519104134 |
| CbMPDiCbraya | duration | 2W | DL | 0 | 0 | 0 | 0 | 0.338109844 | 0 |
| CbMPDiCbraya | duration | 2W | HD | 0.131393498 | 0.020973651 | 0 | 0.007090498 | 0 | 0.016738522 |
| CbMPDiCbraya | duration | 2W | DR | 0.862264518 | 0.441095114 | 0.612198606 | 0.169051612 | 0.599586566 | 0.464157344 |
| CbMPDiCbraya | duration | 2W | DominantProcess | DR | HoS | DR | HoS | DR | HoS |
| CbMPDiCbraya | duration | 2W | DominantProcessImportance | 0.862264518 | 0.537931234 | 0.612198606 | 0.82385789 | 0.599586566 | 0.519104134 |
| CbMPDiCbraya | duration | 2W | DominantProcessPvalue | 0.072 | 0.401 | 0.136 | 0.004 | 0.213 | 0.483 |
| CbMPDiCbraya | duration | 4W | HeS | 0 | 0 | 0.124102319 | 0 | 0.03437464 | 0 |
| CbMPDiCbraya | duration | 4W | HoS | 0.309246044 | 0.211890338 | 0.071167737 | 0.796519908 | 0.0339908 | 0.646222313 |
| CbMPDiCbraya | duration | 4W | DL | 0.387795514 | 0 | 0 | 0 | 0 | 0 |
| CbMPDiCbraya | duration | 4W | HD | 0.011521545 | 0.038144542 | 0.010739196 | 0.026824489 | 0 | 0.049450882 |
| CbMPDiCbraya | duration | 4W | DR | 0.291436897 | 0.74996512 | 0.793990748 | 0.176655603 | 0.93163456 | 0.304326804 |
| CbMPDiCbraya | duration | 4W | DominantProcess | DL | DR | DR | HoS | DR | HoS |
| CbMPDiCbraya | duration | 4W | DominantProcessImportance | 0.387795514 | 0.74996512 | 0.793990748 | 0.796519908 | 0.93163456 | 0.646222313 |
| CbMPDiCbraya | duration | 4W | DominantProcessPvalue | 0.562 | 0.012 | 0.016 | 0.017 | 0 | 0.154 |
| CbMPDiCbraya | duration | 6W | HeS | 0 | 0 | 0 | 0 | 0.035192366 | 0 |
| CbMPDiCbraya | duration | 6W | HoS | 0.228389618 | 0.418368155 | 0.039547584 | 0 | 0.118958443 | 0.803698384 |
| CbMPDiCbraya | duration | 6W | DL | 0.239914069 | 0.203630027 | 0.01997465 | 0.434928475 | 0.26686681 | 0.10618378 |
| CbMPDiCbraya | duration | 6W | HD | 0.032458571 | 0.01037799 | 0.00471254 | 0.005388977 | 0 | 0.01474889 |
| CbMPDiCbraya | duration | 6W | DR | 0.499237742 | 0.367623827 | 0.935765226 | 0.559682548 | 0.578982381 | 0.075368947 |
| CbMPDiCbraya | duration | 6W | DominantProcess | DR | HoS | DR | DR | DR | HoS |
| CbMPDiCbraya | duration | 6W | DominantProcessImportance | 0.499237742 | 0.418368155 | 0.935765226 | 0.559682548 | 0.578982381 | 0.803698384 |
| CbMPDiCbraya | duration | 6W | DominantProcessPvalue | 0.305 | 0.512 | 0 | 0.37 | 0.095 | 0.007 |

Table S4 Keystone species in the different treatments.

| Treatments | Category | Taxa |
| --- | --- | --- |
| **NH** |  |  |
| SH183636.07FU_EF126342_reps | module hub | k__Fungi;p__Mortierellomycota;c__Mortierellomycetes;o__Mortierellales;f__Mortierellaceae;g__Mortierella |
| SH200142.07FU_KU561825_reps | module hub | k__Fungi;p__Ascomycota;c__Sordariomycetes;o__Microascales;f__Ceratocystidaceae;g__Ceratocystis |
| SH202944.07FU_HG328072_reps | Connector | k__Fungi;p__Ascomycota;c__Sordariomycetes |
| SH209070.07FU_EF568081_refs | module hub | k__Fungi;p__Ascomycota;c__Sordariomycetes;o__Hypocreales;f__Nectriaceae;g__Nectria;s__unidentified |
| **LH** |  |  |
| SH195293.07FU_HM365253_refs | module hub | k__Fungi;p__Ascomycota;c__Sordariomycetes |
| SH198997.07FU_KR232527_reps | module hub | k__Fungi;p__Ascomycota |
| **HH** |  |  |
| SH195314.07FU_KP204017_refs | module hub | k__Fungi;p__Ascomycota;c__Sordariomycetes;o__Hypocreales;f__Nectriaceae;g__Nectria;s__unidentified |
| **2W** |  |  |
| SH176583.07FU_FN397265_reps | Connector | k__Fungi;p__Ascomycota;c__Sordariomycetes;o__Hypocreales;f__Nectriaceae;g__Fusarium;s__Fusarium_solani |
| SH183633.07FU_JX975906_reps | Connector | k__Fungi;p__Mortierellomycota;c__Mortierellomycetes;o__Mortierellales;f__Mortierellaceae;g__Mortierella |
| SH190979.07FU_HG935585_reps | Connector | k__Fungi;p__Ascomycota |
| SH195297.07FU_AJ390388_refs | Connector | k__Fungi;p__Ascomycota;c__Sordariomycetes |
| SH195314.07FU_KP204017_refs | Connector | k__Fungi;p__Ascomycota;c__Sordariomycetes;o__Hypocreales;f__Nectriaceae;g__Nectria;s__unidentified |
| SH198997.07FU_KR232527_reps | Connector | k__Fungi;p__Ascomycota |
| SH221744.07FU_UDB017890_refs | Module hub | k__Fungi;p__Basidiomycota;c__Agaricomycetes |
| **4W** |  |  |
| None |  |  |
| **6W** |  |  |
| SH213730.07FU_KU702674_reps | Module hub | k__Fungi;p__Ascomycota |
| SH198997.07FU_KR232527_reps | Module hub | k__Fungi;p__Ascomycota |
